# The Effect of LAB as Probiotic Starter Culture and Green Tea Extract Addition on Dry Fermented Pork Loins Quality

**DOI:** 10.1155/2015/452757

**Published:** 2015-04-19

**Authors:** Katarzyna Neffe-Skocińska, Danuta Jaworska, Danuta Kołożyn-Krajewska, Zbigniew Dolatowski, Luiza Jachacz-Jówko

**Affiliations:** ^1^Faculty of Human Nutrition and Consumer Sciences, Warsaw University of Life Sciences (WULS), 159C Nowoursynowska Street, 02-776 Warszawa, Poland; ^2^Faculty of Food Sciences and Biotechnology, University of Life Sciences in Lublin, 8 Skromna Street, 20-704 Lublin, Poland

## Abstract

The objective of this study was to evaluate the microbiological, physicochemical, and sensory quality of dry fermented pork loin produced with the addition of *Lb. rhamnosus* LOCK900 probiotic strain, 0.2% glucose, and 1.5% green tea extract. Three loins were prepared: control sample (P0: no additives), sample supplemented with glucose and probiotic strain (P1), and sample with glucose, green tea extract, and probiotic (P2). The samples were analyzed after 21 days of ripening and 180 days of storage. The results indicated that the highest count of LAB was observed both in the samples: with probiotic and with probiotic and green tea extract (7.00 log cfu/g after ripening; 6.00 log cfu/g after storage). The oxidation-reduction potential values were lower in the probiotic loin samples. Probiotic and green tea extract have not caused color changes of study loins during storage. The study demonstrated that an addition of probiotic and green tea extract to dry fermented loins is possible and had no impact on sensory quality after product storage.

## 1. Introduction

Probiotic strains, mainly lactic acid bacteria (LAB) and bifidobacteria, are defined as live microorganisms that have a beneficial impact on humans or animals by improving their intestinal microbial balance. Probiotic bacteria cause a health effect on the host when the number of live bacteria cells in foodstuff is between 6.00 and 8.00 log cfu/g. Dairy products such as yogurt, cheese, ice cream, and others are considered the best vehicle for delivering probiotic strains to the human gastrointestinal tract. Probiotic bacteria could be also used in other food products, including dry fermented meat [1–4].

Dry fermented pork loins are one of the most valuable foodstuffs. No other technologies like heating or mild heating and no chemical antioxidants additions provide such a product rich in a variety of bioaroma and bioflavour compounds. The specific quality is obtained during the fermentation and in the ripening process due to reaction of metabolism of probiotic starter cultures. The available science literature presents increasing number of information about the possibility of the use of probiotic starter culture in dry fermented sausage [3, 5–7], although Kołożyn-Krajewska and Dolatowski [8] in a wide review article summarized researches which show that probiotic starter cultures can be used for production, not only dry fermented sausages but also hams and loins. However probiotic starter cultures are not widely used in meat industry. Manufacturing probiotic meat products is much more difficult than that of other probiotic products and such products are at their initial development stage. Probiotic strains must be well adapted to the conditions prevailing in dry fermented meat products. For example, meat is a poor source of simple sugars which are necessary for proper lactic acid fermentation. An addition of monosaccharide at 0.4 to 0.8% improves the growth and survival of LAB, including probiotic strains. Further meat products are characterized by their own natural microflora, which includes LAB. Probiotic bacteria strains that can be used in the manufacturing of fermented meat products should survive in fermented products and they should dominate other microorganisms found in the product [9, 10].

Probiotic starter cultures play an important role during fermentation, ripening, and storage of meat products, increasing their health promoting properties and improving their microbiology and sensory quality, but on the other hand it can accelerate lipid oxidation, as a result making the shelf life shorter [3, 11]. Lipid oxidation in muscle food can be controlled with the use of synthetic or natural antioxidants. Recently, the functional properties of plant extracts have been investigated due to their potent antioxidant and nutraceutical activity. Tea catechins constitute a major group of polyphenolic flavonoids found in green tea. The antioxidant properties of tea catechins have been demonstrated in a variety of test systems, also in beef, pork, and poultry. Zhang et al. [2] reported that crude tea catechins were found to be more effective in reducing lipid oxidation than *α*-tocopherol or BHA. Metal ions would affect antioxidative activity of catechins by their binding to the catechins. Catechins react with metal ions to form metal complexes. The main role of tea catechins in lipid/emulsion systems is to retard lipid oxidation, extend shelf life of the product, and stabilize emulsion systems [12]. Therefore lipid oxidation can cause changes in the quality parameters of meat, such as color, taste, aroma, texture, or nutritional value, and therefore it is the primary cause of quality deterioration in meat products. The shelf life of fermented meat products is not determined by bacterial deterioration but by physical and chemical spoilage, including lipid oxidation [2, 10–13].

The aim of the study was to investigate the growth and survival of* Lactobacillus rhamnosus* LOCK900 probiotic starter culture in dry fermented pork loins produced with the addition of green tea extract in the aspect of selected physicochemical properties and sensory quality after 21 days of ripening and after 180 days of vacuum storage.

## 2. Materials and Methods

### 2.1. Preparation of the Probiotic Starter Culture

Probiotic strain* Lactobacillus rhamnosus* LOCK900 (formerly* Lactobacillus casei* ŁOCK 0900; patent number 209988) was used in the study. The strain of probiotic bacteria was originally isolated from feces of a healthy 26-year-old woman and obtained from the Pure Culture of the Technical University, Łódź, Poland [14].

*Lb. rhamnosus* LOCK900 fulfills the criteria required for probiotic bacteria [14, 15]. Strain LOCK900 was selected on the basis of results of in vitro and in vivo studies on animals, which comprised determination of resistance to the acidity of gastric juice and to bile, adherence to epithelial cells, and antimicrobial activity. This strain showed strong antagonistic activity against gram-positive and gram-negative pathogens [16]. Furthermore* Lb. rhamnosus* LOCK900 was isolated among the typical microflora of human intestines and therefore can be safely used for the production of fermented foodstuffs.

Sterile selective culture medium (broth MRS-Mann, Rogosa, Sharpa; growth medium, BIOKAR DIAGNOSTICS, Beauvais, France) and sterile foodstuff were used to prepare starter probiotic cultures. The process of preparing the starter cultures of probiotic strain included two stages.

- Boost process: 5 mL of MRS broth was inoculated with probiotic strain and incubated for 24 h at 37°C.
- Preparation process of starter probiotic cultures: after 24 h the tubes were centrifuged (10000 ×g/5 min; laboratory centrifuge MPW-251; MPW MED. INSTRUMENTS, Warsaw, Poland) to separate the cells of* Lb. rhamnosus* LOCK900 from growth medium MRS. MRS broth was replaced with a foodstuff medium to allow further growth of probiotic bacteria for 24 h at 37°C (subject of patent).

In the prepared starter cultures the number of probiotic bacteria was determined by a Tempo System (precise procedure for determining the number of lactic acid bacteria is described in subsection “Microbiological analyses”). The count of probiotic bacteria was approximtely 9.00 log cfu/mL.

### 2.2. Preparation of the Green Tea Extract

The dried tea leaves “Formosa Lung Ching” (4 g) were poured with the water at a temperature of 85°C (100 mL) and then infused for 5 minutes. After this time the extract was filtered with the tea strainer and cooled to a temperature of 4°C. Green tea extract was added to the surface of loins.

### 2.3. Production Process of Dry Fermented Pork Loins

Pork loins, a strain of probiotic bacteria* Lb. rhamnosus* LOCK900, glucose, and green tea extract “Formosa Lung Ching” were used in the production of dry fermented meats. The meat was chilled to 7 ± 1°C and divided into parts 48 hours after death. Loins samples were cured using “dry” method with the curing mixture (20 g of NaCl; 9.7 g of curing salt; and 0.3 g of NaNO_3_ per kg of loin) in the quantity of 2.5%. The curing process was conducted for 72 h in 0 ± 1°C. Then the strain* Lb. rhamnosus* LOCK900 (2% per kg of meat, containing approx. 9.00 log cfu/mL), green tea extract (1.5% per kg of meat), and glucose (0.2% per kg of meat) were added to the samples of loins. Subsequently, the loins were fermented at 16–18°C for 21 days in fermentation chamber with a relative humidity between 80 and 90%. After 10 days the loins were cold smoked for an hour. After 21 days of ripening the samples were vacuum-packed and stored at 4 ± 1°C for 180 days. Microbiological, physicochemical, and sensory analyses were done after 21 days of ripening and 180 days of storage.

Three different kinds of loins samples were produced: control fermented samples without any additives (P0), fermented samples with an addition of glucose and bacteria strain* Lb. rhamnosus* LOCK900 (P1), and fermented samples with an addition of glucose, bacteria strain* Lb. rhamnosus* LOCK900, and green tea extract (P2). Three series of experiments were performed.

### 2.4. Microbiological Analyses

The aim of the microbiological analysis was the identification of the number of LAB included probiotic bacteria (log cfu/g) in the samples ripened for 21 days and samples stored for 180 days. The analyses were carried out using the Tempo System (automated quality indicator solution; bioMérieux, Mercy Etoile, France) and Tempo LAB tests (automated test for the enumeration of LAB microorganisms; bioMérieux, Mercy Etoile, France). The calculation of bacteria number in the study samples (log cfu/g), according to the Tempo System, is based on the most probable number (MPN) method. Tempo LAB test was allowed to obtain performance levels similar to the standard NF ISO 15214: 1998 [17]. The dilution of the sample was 1/400 in a single vial. The inoculated medium was moved into the Tempo card by Tempo Filler. The cards were incubated for 48 hours at 37°C.

### 2.5. Physicochemical Analyses

The pH of the loin homogenate was measured with a digital pH meter CPC-501 (Elmetron, Zabrze, Poland) equipped with combined electrode ERH-111 (PN-ISO 2917:  2001).

Oxidation-reduction potential (ORP) of the loin homogenate was determined according to the method of Nam and Ahn [18] with slight modification. ORP measurements of the homogenates were carried out using pH meter set to the millivolt scale and equipped with redox electrode.

Lipid oxidation was determined as thiobarbituric acid reactive substances (TBARS) values following the procedure of Pikul et al. [19]. Intensity of color produced in the reaction of malondialdehyde with 2-thiobarbituric acid was measured by means of Nicole Evolution 300 spectrophotometer (Thermo Electron Corporation, Marietta, OH, USA) at a wave length of 532 nm. The values of TBARS were expressed as milligram of malondialdehyde (MDA) per kg of the sample.

Color parameters (CIE *L* ^∗^ *a* ^∗^ *b* ^∗^) were taken immediately after the samples were prepared using 8200 Series reflection spectrocolorimeter (X-Rite, Grand Rapids, MI, USA) with a D65 illuminant and 10° standard observed. Prior to use, the spectrocolorimeter was calibrated against white and black standard. An index Δ*E* ^∗^ describing the total color change of samples over 180 days of storage was calculated using the following formula:

(1) $$\begin{array}{c} \Delta E^{*}={\left[{\left(\Delta L^{*}\right)}^{2}+{\left(\Delta a^{*}\right)}^{2}+{\left(\Delta b^{*}\right)}^{2}\right]}^{0.5}. \end{array}$$

Each sample was analyzed three times.

### 2.6. Sensory Analyses

For sensory assessment, the sensory QDA method was used [20]. Descriptors were chosen and defined during a panel discussion and then verified in a preliminary session. Finally, 16 sensory attributes were measured to quantify the quality of the tested products (Table 1).

An unstructured, linear graphical scale of 100 mm is afterwards converted to numerical values (0–10 conventional units (c.u.)). The marks of anchors of the tested attributes were for most of them as follows: none-very strong, for meat colour: light-dark, for homogeneity of colour: none-very high, and for juiciness: dry-juicy. On the basis of the abovementioned quality characteristics, the assessing sensory panel indicated an overall sensory quality (low-very high) for each sample on a separate scale.

Meat samples were sliced into approximately equal size weight (ca. 10 g) and placed in plastic, odourless, disposable boxes (volume 125 mL) covered with lids. Sensory panel (10-person) was formally trained [21] and proper experienced (3–8 years of sensory evaluation practice).

### 2.7. Statistical Methods

The microbiological, physicochemical, and sensory results were analyzed using a one-way analysis of variance (ANOVA). Further the results of microbiological evaluation were analyzed using a linear regression and a correlation to check the influence of 21 days of ripening time and of 180 days of storage time for growth of LAB at dry fermented pork loins.

The significance of the differences between mean values was calculated at the significance level of *P* < 0.05 and in the case of sensory analysis at the significance level of *P* < 0.01, using Tukey's range test. Results of Quantitative Descriptive Analysis (QDA) were also analyzed by principal component analysis (PCA) performed by the use of Analsens NT software (Polish Academy of Sciences). All statistical analyses (except PCA) were calculated using Excel 6.0 (Microsoft, Bloomington, IL, USA), Statistica 8.0 (StatSoft, Inc.).

## 3. Results and Discussion

### 3.1. Microbiological Analyses

The available science literature generally presents information about growth and survival of probiotic bacteria in dry fermented sausages [9, 10, 22–25]. There is no detailed information about the possibilities of using probiotic starter cultures with green tea extract to production dry fermented loins or hams.

In our previous study it was shown that potential probiotic starter cultures had a good ability to survive in dry fermented pork loins and can be used for cured loin production. Also, it was indicated that probiotic starter cultures* Lb. rhamnosus* LOCK900 and LOCK908 added to dry fermented pork loins grew and survived a count of 7.00 log cfu/g in samples without and 8.00 log cfu/g in samples with 0.2% glucose addition. In the subsequent study it was demonstrated that optimal fermentation conditions (21 days of ripening in 20°C) allowed producing dry fermented loins with a high count of lactic acid bacteria including probiotic strain* Lb. rhamnosus* LOCK900 (7.00-8.00 log cfu/g), persisting during 180 days of vacuum storage.

**Figure 1:**
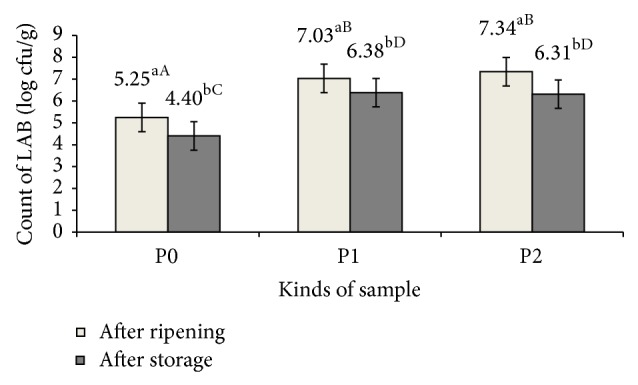
Microbiology evaluation of the fermented pork loins produced with probiotic culture* Lb. rhamnosus* LOCK900 and glucose (P1),* Lb. rhamnosus* LOCK900 with glucose and extract of green tea (P2), and the controls (P0) after 21 days of ripening and after 180 days of storage. Means followed by the same lower case letters within columns and capital letters within row are not significantly different (*P* < 0.05).

The present results of the microbiological analyses, after ripening (21 days, 18 ± 1°C) and after 180 days of chilling storage under anaerobic conditions, was to assess the effect of green tea extract addition, natural antioxidant, on the growth and survival of probiotic strain* Lb. rhamnosus* LOCK900 (Figure 1).

The number of LAB in control samples (P0), without probiotic bacteria or glucose and green tea extract addition, was at the level of approximately 5.00 log cfu/g after ripening and 4.00 log cfu/g after storage time. In the case of samples with glucose addition (P1) and both glucose and green tea extract addition (P2), including probiotic bacteria* Lb. rhamnosus* LOCK900, a much better growth and survival of LAB were observed as compared to control. The total count of LAB in these samples was approximately 7.00 log cfu/g after ripening process and 6.00 log cfu/g after storage time.

The statistical analysis of the microbiological results showed that time of storage (180 days at the 4 ± 1°C) significantly affected the survival of LAB in loin samples (Figure 1). However, dry fermented loins with the addition of probiotic strains* Lb. rhamnosus* LOCK900 (P1 = 7.03–6.38 log cfu/g and P2 = 7.34–6.31 log cfu/g; *P* < 0.05) were characterized by higher count of lactic acid bacteria after 6 months of vacuum storage than controls (P0 = 5.25–4.40 log cfu/g; *P* < 0.05). Obtained differences in the number of LAB between the dry fermented loins without (control-P0) and with the addition of* Lb. rhamnosus* LOCK900 (P1 and P2) indicated a good growth of probiotic strain during three weeks of ripening process and good survival for all time during storage.

Lactic acid bacteria, including probiotic strains, are main agents of meat fermentation improving microbiological, sensory, and physicochemical quality of the final product [7, 26]. Tea catechins have been used as antioxidant compounds in many food matrices such as fishes, poultries, and meat like antibacterial, antifungal, and antiviral agents [2, 27]. However, in the global literature there are no researches which used probiotic starter cultures and green tea extract addition to produce dry fermented meat. The results obtained in our experiment indicated that application of green tea extract had no negative effect on growth and survival of lactic acid bacteria, including probiotic strain during the ripening process and half-year vacuum storage. The count of LAB, including* Lb. rhamnosus* LOCK900, remained at the same level as in the sample with only glucose addition (P1 and P2 = approx. 7.00 log cfu/g after production and 6.00 log cfu/g after storage).

Also, the studies of López de Lacey [28] have demonstrated that green tea does not affect the growth of probiotic strains, like a* Lactobacillus paracasei* L26 and* Bifidobacterium animalis *spp.* lactis* B94. In Jaziri et al. [29] research it has been also shown that addition of green tea extract did not influence the survival of characteristic microorganisms, mainly* Streptococcus thermophilus* and* Lactobacillus bulgaricus*, in yogurts during production process and 6 weeks of storage. According to the previous studies, Lee et al. [30] and McCue and Shetty [31] also demonstrated that LAB such as* Lactobacillus* spp. were not affected by tea antioxidants compounds in opposition to pathogenic microorganisms. It can be stated that natural antioxidants contained in green tea extract have antibacterial ability effect only on pathogenic microorganisms [27, 32]. Kachouri and Hamidi [33] and Vermeiren et al. [34] observed that LAB, including probiotic strains, could have a synergistic antioxidant and nitrite effect of inhibiting pathogenic bacteria, for example,* Listeria monocytogenes* and* Clostridium botulinum*. On the other hand, Wójciak et al. [35], Min and Ahn [36], and also Talon et al. [37] found that the probiotic bacteria have significantly affected the oxidation and reduction potential of cured pork meat products. Probiotic bacteria and natural antioxidants could be an alternative way of maintaining a high nutritional and flavour quality of meat products without the use of conventional methods of food preservatives [26, 32, 38].

### 3.2. Physicochemical Analyses

Most publications are concerned with the growth and survival of probiotic bacteria added to dry fermented meat products and with their sensory quality. However the shelf life of dry fermented meat products is not entirely limited by bacterial deterioration but by physical and chemical changes [3, 8]. The results of the physicochemical changes in the study dry fermented loins with probiotic strain and green tea extract, after 21 days of ripening and after 180 days of chilling storage under anaerobic conditions, are presented in Table 2.

Dry fermented pork loins with an addition of probiotic bacteria and glucose (P1) were characterized by the highest acidity compared to the other samples, both after production and after storage. The acidity of all loin samples significantly increased during the storage period. The control samples (P0) and the samples with addition of probiotic bacteria, glucose, and green tea extract (P2) were characterized by similar pH values during the whole period of storage (5.26-5.27). Lower pH value of sample P1 (5.04) could be a result of organic acids production by bacteria, mainly lactic acid resulting in the acidification of the environment. Also in Skwarek and Dolatowski [39] research it has been shown that addition of probiotic strain* Lb. rhamnosus* LOCK900 and glucose to dry fermented hams has an effect on the hams acidity increase during ripening. Herrero et al. [40] found that low pH value inhibits the saprophytic and pathogenic microflora growth in meat products.

Statistical analysis indicated that the use of probiotics, glucose, or glucose and green tea extract in the production of dry fermented loins caused no significant effect on oxidation-reduction potential values after ripening and the refrigeration storage. Oxidation-reduction potential (ORP) values of the samples P1 and P2 ripened for 21 days were lower compared to the control sample. Simultaneously the lowest value of oxidation-reduction potential observed in sample P2 after ripening (317.77 mV) could be evidence of system tendency to inhibit the oxidation process and effective use of natural antioxidants in the green tea extract. Redox potential of the samples P0 and P2 increased during the storage. The ORP values of variants of experimental samples were similar alike after storage (approximately 335 mV). The lower ORP value (322.27 mV) was observed in the case of the sample P1 containing strain* Lb. rhamnosus* LOCK900 and glucose. Tea catechins are an efficient free radical scavenger due to their one electron reduction potential. Antioxidant activity as hydrogen or electron donors is determined by this reduction potential of free radicals. A lower reduction potential has a tendency to lose electron or hydrogen. The rate of reaction with free radicals and the stability of the resulting antioxidant radicals contribute to the reactivity of antioxidant [36].

After ripening TBARS values were significantly higher for the samples P1 and P2 (~1.0 mg MDA/kg sample) than for the control sample P0 (0.37 mg MDA/kg sample). A higher TBARS value in the samples P1 and P2 may show the catalytic activity of probiotic bacteria on the oxidative processes of lipids as a result of hydrogen peroxide production by some strains [41]. The mentioned parameter significantly increased after the storage only in the case of control sample. Addition of* Lb. rhamnosus* LOCK900 and green tea extract inhibited lipids oxidation. After the storage period the lowest value of the oxidation rate, equal to 0.64 mg MDA/kg of the sample, was reported for the sample P1. Low TBARS value of the sample P1 may be due to inhibition of activity of the bacteria responsible for lipid oxidation and conversion. Bozkurt [12] showed similar ratio of TBARS values in the sausage with an addition of green tea extract. He also found that the use of tea antioxidants can inhibit the growth of bacteria, mainly* Enterobacteriaceae*, responsible for the formation of biogenic amines. Min and Ahn [36] had suggested that meat products with low pH value had generally strong prooxidant effect. In our study pH decreasing in meat products during storage had affected the increase of lipid oxidation (TBARS value) only in the case of sample without any additives (P0).

Significant differences in colour parameters *L* ^∗^, *a* ^∗^, and *b* ^∗^ between the samples were noted only after the storage (Table 2). Storage time had a significant effect (*P* < 0.05) on *a* ^∗^ and *b* ^∗^ values only in the case of meat samples with addition of the strain* Lb. rhamnosus* LOCK900 and glucose (P1). The studies found out a significant (*P* < 0.05) increase of the value of parameter *L* ^∗^ (52.67) of colour in the samples with an addition of probiotic bacteria and glucose (P1) after storage as compared to the control samples, whereas the highest proportion of yellow colour (*b* ^∗^ = 7.96) after the storage was observed in the control samples, significantly (*P* < 0.05) higher *b* ^∗^ value than the samples with an addition of probiotic bacteria, glucose, and green tea extract (*b* ^∗^ = 5.25). Possibly an increase of the value of *L* ^∗^ parameter for the sample P1 after 180 days of storage was associated with the higher acidity pH decrease, due to lactic acid accumulation. Lipid oxidation could have an influence on the *L* ^∗^ value of meat product. Decrease of the value of the parameter *b* ^∗^ may be a result of the lactic acid bacteria growth during meat products ripening. Lowering amount of oxygen affects the decrease of oksymyoglobin and then the decrease of *b* ^∗^ value.

According to Pérez-Alvarez et al. [42] lower value of parameter *b* ^∗^ is due to decreasing of the concentration of myoglobin and/or oksymyoglobin as they react with nitrite to nitrosylmyoglobin. The values of *a* ^∗^ parameter of all experimental loins samples increased during the storage period, but significant (*P* < 0.05) differences were noted only in the case of sample P1. Salejda et al. [43] also observed a tendency to increase the value of *a* ^∗^ parameter during storage of model meat products with green tea extracts. The highest value of *a* ^∗^ parameter (significantly higher than value of *a* ^∗^ parameter for the samples P0 and P2) was observed in the sample P1 (*a* ^∗^ = 7.69) after the storage. Higher values of *a* ^∗^ could be due to increased content of created nitrosylmyoglobin.

Analysis of total color change Δ*E* ^∗^ showed no effect of strain* Lb. rhamnosus* LOCK900, glucose, or glucose and green tea extract on meat product color stability during the storage. There were no significant differences of Δ*E* ^∗^ (*P* < 0.05) between all experimental meat products during storage.

### 3.3. Sensory Analyses

The results obtained in this study indicated that an application of glucose and probiotic strains or application of glucose and a bacteria strain* Lb. rhamnosus* LOCK900 as well as green tea extract affected overall sensory quality of tested samples. The highest sensory quality after ripening was obtained in the case of fermented pork loin samples without additives. The samples with glucose and probiotic strains or with glucose, a bacteria strain, and green tea extract were characterized by lower sensory quality. The decrease of overall sensory quality in case of samples P1 and P2 was related to decreasing the intensity of attributes which had positive impact on sensory quality, like smoked odour and flavour as well as dried meat odour and increasing the negative attributes like “off-flavor” and “others” (mostly described by panellists as “acid”).

The results obtained after storage time indicated that the overall sensory quality regarding three tested samples was comparable. The intensity of tested attributes did not differ from the individual samples. The study indicated also that addition of probiotic or probiotic and green tea extract had no impact on sensory quality after product storage rather the storage time of 180 days decreased the overall quality of all tested samples in comparison to their quality after ripening.

**Figure 2:**
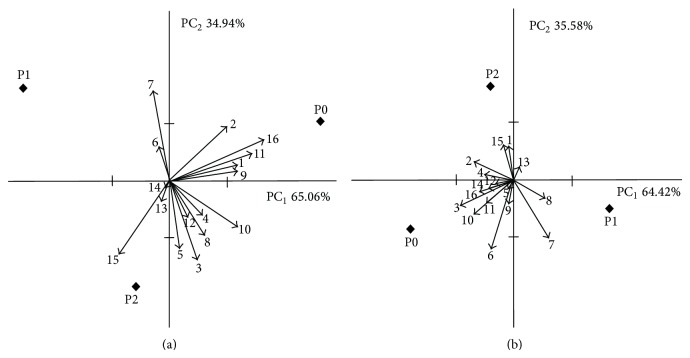
(a), (b) Principal components analysis of the matrix of mean sensory attributes ratings across the fermented pork loins produced with probiotic culture* Lb. rhamnosus* LOCK900 (P1),* Lb. rhamnosus* LOCK900 with glucose and extract of green tea (P2), and the control (P0); (a) after ripening; (b) after storage. (1) Smoked odour, (2) dried meat odour, (3) acid odour, (4) off-flavour odour, (5) other odours, (6) colour intensity, (7) colour homogeneity, (8) juiciness, (9) smoked flavor, (10) dried meat flavor, (11) salty taste, (12) bitter taste, (13) stinging flavor, (14) storage flavor, (15) other flavor, and (16) overall quality.

The magnitude and type of differences and similarities of the tested fermented pork loin samples are shown in the PCA presentation (Figures 2(a) and 2(b)). In the case of ripened samples, vector directions of smoked odour and flavour (vector 1 and 9) and dried meat odour and flavour (vector 2 and 10) versus overall sensory quality (OSQ)-vector (vector 16) allows the conclusion to be drawn that they were positively correlated with OSQ. Mainly “other” flavours (vector 14), storage flavor (vector 14), and stinging (vector 13) had a negative impact on OSQ of the tested samples (Figure 2(a) and 2(b)). Closing position of sample P0 to the vector 16 (overall quality) indicated the highest quality of sample P0 after ripening and the quality is described mostly by smoked odour and flavour as well as dried meat odour and flavour (Figure 2(a)). The position of sample P1 indicated their characteristic by colour attributes. The sample P2 was characterized by “other” odours and flavour (Figure 2(a)). The PCA presentation of quality of storage samples (length and position of vectors) confirms the similarity of the tested samples (Figure 2(b)).

Our previous study indicated that an application of potential probiotic strains tested in this research did not significantly affect overall sensory quality of ripened samples. A negative side effect of probiotic culture on the overall quality of the fermented meat was indicated in the paper of De Vuyst et al. [44]. Furthermore in Zdolec et al. [45] research it has been also shown that addition of probiotic culture of* Lactobacillus sakei* for fermented sausages did not have a negative impact on the sensory properties of the sausages and in case of chosen sensory parameters. The authors [45] observed improvement, mainly in acidity and tenderness. In case of green tea extract addition results of other studies [12] indicated that pH, colour, and overall sensory quality were not affected by the addition of green tea extract in fermented sausages during 15 days of ripening, whereas, the study of Valencia et al. [46] showed that sensory scores for parameters such as colour, texture, flavour, and overall acceptability were not significantly affected (*P* > 0.05) by the addition of green tea catechins. Similar results were obtained in Mitsumoto et al. [13] study where tea catechins treatments resulted in no significant differences in the sensory flavour and tenderness of cooked meat compared to controls treatments. Tea catechins treatments had no effects on overall acceptability of cooked beef patties, whereas decreased acceptability of chicken patties.

## 4. Conclusion

The present microbiological study demonstrated a much better growth and survival of LAB in the case of samples with glucose addition and both glucose and green tea extract addition with probiotic bacteria* Lb. rhamnosus* LOCK900. Probiotic starter culture and green tea extract have not caused color changes of dry fermented pork loins during storage. The oxidation-reduction potential values of the tested samples ripened were lower compared to the control sample. Simultaneously, an addition of probiotic strain* Lb. rhamnosus* LOCK900 and green tea extract in the production of dry fermented loins had a positive effect in inhibiting lipid oxidation of meat products during storage. Lower pH values of meat products during storage increased lipid oxidation only in the sample without any additives. Regarding sensory analysis, an addition of probiotic or probiotic and green tea extract had no impact on sensory quality after product storage. The storage time decreased the overall quality of all tested samples in comparison to their quality after ripening.

It can be concluded that the probiotic strain* Lb. rhamnosus* LOCK900 and green tea extract can be used in the production of dry fermented pork loins with good sensory, physicochemical quality and high number of bacteria.

## Figures and Tables

**Table 1:** Definition of the sensory attributes.

Attribute	Definition
ODOUR	
Smoked meat	Typical of dry fermented meat
Dried meat	Typical of dry fermented meat
Sharp	Irritating impression when smelling
Rancid	Off-flavour associated with changes in fat oxidation; lack of freshness
Other	Other sensations, out of the list
APPERANCE and TEXTURE	
Meat colour	Intensity of the red colour associated to the meat
Homogeneity of colour	Uniform distribution of red colour typical of dry fermented meat
Juiciness	Perception of the amount of water released by the product during mastication
FLAVOUR	
Smoked meat	Typical of dry fermented meat
Dried meat	Typical of dry fermented meat
Salty	Basic quality of taste
Bitter	Basic quality of taste
Stored	Lack of freshness
Stinging	Basic quality of taste
Sour	Basic quality of taste
Other Overall quality	Other sensations, out of the list Attribute of total quality of dry fermented pork loin

**Table 2:** Assessment of physicochemical quality of dry fermented loins (means ± standard deviations).

Variants/storage time (days)	Kinds of samples		
	P0	P1	P2
pH			
0, after ripening	5.53 ± 0.05^A^	5.41 ± 0.18^C^	5.61 ± 0.01^E^
180	5.27 ± 0.02^aB^	5.04 ± 0.03^cbD^	5.26 ± 0.02^dF^
ORP [mV]			
0, after ripening	331.43 ± 20.88	324.90 ± 1.23	317.77 ± 0.90
180	334.30 ± 20.03	322.37 ± 34.96	335.37 ± 16.96
TBARS [mg MDA/kg]			
0, after ripening	0.37 ± 0.00^acA^	1.04 ± 0.01^bC^	1.00 ± 0.01^dE^
180	0.74 ± 0.19^B^	0.64 ± 0.19^D^	0.72 ± 0.23^E^
Colour parameters			
*L* ^∗^			
0, after ripening	50.11 ± 1.64	48.34 ± 2.80^a^	51.89 ± 2.37^a^
180	46.08 ± 2.34^a^	52.67 ± 1.31^b^	46.40 ± 3.59
*a* ^∗^			
0, after ripening	5.60 ± 2.19	4.64 ± 0.38^B^	4.24 ± 0.03
180	5.83 ± 0.75^a^	7.69 ± 0.51^bcC^	5.55 ± 0.84^d^
*b* ^∗^			
0, after ripening	7.12 ± 0.64	5.42 ± 0.91^B^	7.26 ± 1.02
180	7.96 ± 1.06^a^	7.80 ± 0.56^cC^	5.25 ± 0.97^bd^
Total change of color Δ*E* ^∗^	4.49 ± 3.97	6.18 ± 2.55	6.59 ± 4.89

^a–d^Means in the same row with different lowercase letters are significantly different (*P* < 0.05).

^A–D^Means in the same column with different capital letters are significantly different (*P* < 0.05).

ORP: oxidation-reduction potential; TBARS: thiobarbituric acid reacting substances; MDA: malondialdehyde; *a* ^∗^: redness value; *b* ^∗^: yellowness value; *L* ^∗^: lightness value.
